# Application of Computed Tomography Angiography in Preoperative Diagnosis of Coarctation of Aorta and Evaluation of Aortic Dilatation in Infants

**DOI:** 10.21470/1678-9741-2023-0160

**Published:** 2024-04-15

**Authors:** Hui-Jun Xiao, Wei-Hua Lin, Shun-Yong Zheng, Yi-Yong Cai

**Affiliations:** 1 Department of Radiology, Zhangzhou Affiliated Hospital of Fujian Medical University, Zhangzhou, People’s Republic of China

**Keywords:** Aortic Coarctation, Thoracic Aorta, Dilatation, Diaphragm, Ventricular Heart Septal Defects, Tomography

## Abstract

**Objective:**

To evaluate the occurrence of aortic dilatation and its associated predictors with coarctation of the aorta (CoA) in infants using multi-slice computed tomography (MSCT).

**Methods:**

The clinical data of 47 infantile patients with CoA diagnosed by MSCT and 28 infantile patients with simple ventricular septal defect were analyzed retrospectively. Aortic diameters were measured at six different levels, and aortic sizes were compared by z score. The coarctation site-diaphragm ratio was used to describe the degree of narrowing. Relevant clinical data were collated and analyzed.

**Results:**

The dilation rate and z score of the ascending aorta in the severe CoA group were significantly higher than those in the mild CoA group (11 [52.38%] vs. 21 [80.77%], P=0.038 and 2.00 ± 0.48 vs. 2.36 ± 0.43, P=0.010). Pearson’s correlation analysis found that the z score of the ascending aorta was negatively correlated with the coarctation site-diaphragm ratio value (r=-0.410, P=0.004). A logistic retrospective analysis found that an increased degree of coarctation was an independent predictor of aortic dilatation (adjusted odds ratio 0.002; 95% confidence interval 0.00-0.819; P=0.043). The z score of the ascending aorta in the severe CoA group was significantly higher than that in the ventricular septal defect group (P<0.05).

**Conclusion:**

Most infants with CoA can also have significant dilatation of the ascending aorta, and the degree of this dilatation is related to the degree of coarctation. Assessment of aortic diameter and related malformations by MSCT can predict the risk of aortic dilatation in infants with CoA.

## INTRODUCTION

**Table:** 

Abbreviations, Acronyms & Symbols				
ANOVA	= Analysis of variance		ECG	= Electrocardiogram
ASIR-V	= Adaptive statistical iterative reconstruction-V		LVEF	= Left ventricular ejection fraction
BAV	= Bicuspid aortic valve		MRI	= Magnetic resonance imaging
BSA	= Body surface area		MSCT	= Multi-slice computed tomography
CDR	= Coarctation site-diaphragm ratio		OR	= Odds ratio
CHD	= Congenital heart diseases		PDA	= Patent ductus arteriosus
CI	= Confidence interval		SBP	= Systolic blood pressure
CoA	= Coarctation of the aorta		SSF	= Snap shot freeze
CT	= Computed tomography		TTE	= Transthoracic echocardiography
CTA	= Computed tomography angiography		VSD	= Ventricular septal defect
DBP	= Diastolic blood pressure			

Coarctation of the aorta (CoA) is one of the most common congenital heart diseases (CHD) and covers about 6-8% of live births with CHD^[1-2]^. As the disease progresses, patients may develop hypertension, left heart failure, aortic dilatation, and even aortic aneurysm, aortic dissection, and aortic rupture^[3]^. According to the survey, 50% of unoperated patients with CoA died before the age of 32 years, and 21% of the cases were caused by aortic aneurysm rupture^[4]^. At the same time, a large cohort of CoA patients typically have a bicuspid aortic valve (BAV), which often goes ahead with ascending aortic dilatation^[5]^. Clinical statistics showed that not only untreated patients with CoA were prone to aortic dilatation, but also patients receiving surgical treatment still had the risk of aortic dilatation^[5]^. The pathogenesis of aortic dilatation is still unclear. Real-time monitoring of the aortic condition of patients with CoA can serve as an early warning. Therefore, it is necessary to evaluate the preoperative aortic diameter of infantile patients with CoA and to explore the related factors affecting aortic dilatation. At present, transthoracic echocardiography (TTE) is a first-line imaging method for the diagnosis of infantile patients with CHD. However, due to the influence of neck length, sternal block, and lung gas interference, the aortic arch cannot be well displayed. At the same time, in infantile patients with CoA, the flow velocity of coarctation can be decreased due to the influence of collateral vessels and patent ductus arteriosus (PDA), so TTE is limited in the evaluation of the aorta^[6]^. Magnetic resonance imaging (MRI) has been widely recognized in this field, especially its advantages of no radiation exposure, but its high cost, long examination time, and low spatial resolution relative to computed tomography (CT) limit its application^[7]^. As a non-invasive imaging technique, CT angiography (CTA) has a high spatial resolution, wide field of vision, short scanning time, and can describe the shape of the aorta in detail. At present, multi-slice CT (MSCT) angiography has been widely used to diagnose and evaluate CHD^[8]^. According to a literature search, there are currently few studies evaluating aortic dilatation and its predictors in infants with CoA by CTA. Therefore, this study aimed to evaluate the occurrence and associated predictors of aortic dilatation in infants with CoA by preoperative CTA.

## METHODS

This study was approved by the ethics committee of our hospital (2022KYB276) in China and followed the principles of the Declaration of Helsinki. Because this was a retrospective study, the requirement of informed consent is exempted. Inclusion criteria were infants diagnosed with CoA by MSCT angiography and who received TTE examination before surgery. Exclusion criteria were as follow: 1. insufficient clinical information; 2. associated with double aortic arch, supra-aortic stenosis, and other aortic malformations; 3. combined with Turner syndrome, Marfan syndrome, Loeys-Dietz syndrome, and mucopolysaccharide storage; 4. combined with aortic compression.

Echocardiography was usually the first choice for CoA; however, in some cases, further cross-sectional imaging was needed to assess aortic development. Both CTA and MRI had excellent performance in this area^[7]^. Our organization preferred CTA as a complementary application for infants because of its high spatial and temporal resolution, complete aortic visualization, short collection time (no sedation is required) and low cost^[8]^. Although iodization contrast load and radiation exposure were important issues, the new dose reduction method significantly reduced the estimated radiation dose while maintaining image quality^[8]^. Therefore, we chose CTA as an important supplementary examination for CHD, especially for infants with vascular malformations.

### Sample Size Calculation

This was a retrospective study, and the subjects’ aortic *z* score was the observed outcome. According to the results of literature review, the mean *z* score of the aorta in the mild CoA group was 1.88, and the mean *z* score of the aorta in the severe CoA group was 2.30, bilateral α=0.05 was set, and the power was 90%. Using PASS 15.0 (NCSS Statistical Software) to calculate the sample size of each group, it was defined as 20 cases. Considering the 10% drop rate, we included 22 samples in each group, for a total of at least 44 subjects.

According to the inclusion and exclusion criteria, 54 infantile patients with CoA treated in our hospital from January 2020 to July 2022 were selected as subjects. All patients underwent routine CTA and TTE before surgery. The basic data such as weight, height, blood pressure (right radial artery pressure), and other clinical examinations were retrospectively collected. Finally, 47 infants with CoA were selected for this retrospective study. At the same time, to further compare the size of the aorta, we selected 28 infants with simple ventricular septal defect (VSD) under the same conditions as the VSD group to compare the aortic dilatation with the CoA group. For patients with VSD who need surgery in infancy, we routinely performed CTA examination before operation to rule out airway and macrovascular malformations. Diagnostic criteria for hypertension for children younger than one year old is made according to the summary table of neonatal blood pressure values compiled by Dionne et al.^[9]^

### Multi-slice Computed Tomography

This study used a GE revolution 256-slice MSCT scanner, combined with 70 kV low tube voltage and prospective electrocardiogram (ECG)-triggered technology. The Revolution CT features a 16 cm wide detector, a rotation of the tube of 0.28 seconds, and snap shot freeze (SSF) algorithm reconstruction technology to significantly improve the time resolution of cardiovascular CT imaging. The SSF technology helps minimize coronary motion artifacts in high heart rate patients. In addition, under the condition of any heart rate or arrhythmia, the whole heart can be scanned by axial scanning within one cardiac cycle, which greatly reduces the cardiac and respiratory motion artifacts, and greatly improves the diagnostic efficiency and image quality of infants with complex CHD^[10]^. The Revolution CT Auto Gating system intelligently recognizes heart rate and rhythm, matching the optimal scan and reconstruction period accordingly to ensure one-beat success. At the same time, the adaptive statistical iterative reconstruction-V (ASIR-V) technology has the advantages of real-time reconstruction and adopts a more advanced system noise model, scanned object model, and physical model, which can further reduce the noise, improve the low density and contrast, and reduce the image artifacts^[11]^. In this study, all the case images can meet the needs of clinical diagnosis, and the CTA images are satisfactory from the perspective of objective evaluation indicators.

### Echocardiographic Measurement

Some studies have shown that vascular dysfunction of the aorta before coarctation is related to CoA-associated hypertension and aortic dilatation^[12]^, so we also included vascular function for the correlation study.

All infants underwent TTE using PHILIPS ultrasound (EPIQ 7C). The cardiac anatomy and function were evaluated by two-dimensional echocardiography, color flow imaging, and continuous wave Doppler. The internal diameter of ascending aorta was measured by two-dimensional M-mode echocardiography in parasternal longitudinal section, 3 cm above the aortic valve level between the trailing edge, and between the anterior and posterior edge of ascending aorta. The aortic stiffness, aortic distensibility, and left ventricular ejection fraction (LVEF) were studied by relevant data. During the whole process of TTE, according to the recommendations of the American Heart Association, an automatic oscilloscope was used to measure supine systolic blood pressure (SBP) and diastolic blood pressure (DBP) three times in the right arm^[13]^. The average value of three measurements was taken to reduce the error, and the difference between SBP and DBP was used as the estimation of the aortic pulse pressure. The accuracy and repeatability of the method had been demonstrated previously^[14]^.

LVEF was determined by modified Simpson’s method from the apical two-chamber section and the apical four-chamber section views using the equation^[15]^. The formulas for calculating distensibility and wall stiffness index came from the relevant literature^[16]^. It has been previously reported that there is a good interobserver agreement and repeatability between observers using this method to measure systolic and diastolic aortic area.

### Computed Tomography Image Analysis

**Fig. 1:**
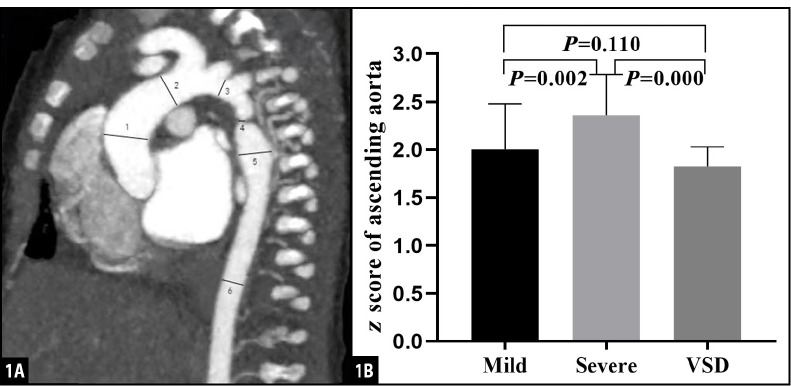
1A) Sagittal multiplanar reformatted image shows the measurement of aortic diameters at different levels: 1=ascending aorta; 2=pre-coarctation aorta; 3=aortic arch; 4=site of coarctation; 5=descending aorta after the site of coarctation; 6=descending aorta at level of diaphragm. 1B) Pairwise comparison of z score of ascending aorta among mild group, severe group, and control group. VSD=ventricular septal defect.

The internal diameter of the aorta at all levels and the concomitant cardiac malformations, including BAV, PDA, VSD, and atrial septal defect was recorded. The images were analyzed by two experienced radiologists using the axial image, maximum density projection, and volume reproduction method. Aortic diameters were measured at six levels: ascending aorta at its maximum diameter (ascending aorta), aorta just proximal to the origin of the brachiocephalic trunk (pre-coarctation aorta), the aortic arch at the largest size (aortic arch), coarctation site at the narrowest size (coarctation site), the widest region of the descending aorta (post-coarctation aorta), descending aorta at the level of the diaphragm, and the length of the coarctation segment of the aorta^[17,18]^ (Figure 1A). The ratio of the aortic diameter at the narrowest point to the horizontal diameter of the diaphragm (coarctation site-diaphragm ratio [CDR]) was calculated to describe the degree of coarctation. CDR < 75% could be diagnosed as CoA^[18]^.Those infantile patients with CoA were classified into two groups based on the severity of coarctation: mild CoA group (CDR > 50%) and severe CoA group (CDR < 50%)^[19]^. Considering the growth-related changes, the aortic diameter was standardized as the *z* fraction, that was, the ratio of the aortic diameter to the square root of the body surface area (BSA). The aortic diameter *z* fraction was determined according to the description of Colan et al.^[20]^ and confirmed by the method described by Dallaire et al.^[21]^ The BSA of all patients was calculated by the DuBois formula^[22]^. Aortic dilatation was defined as the diameter of the main artery *z* score > 2.0^[20,21]^. Age, sex, degree of coarctation, CoA complexity, hypertension, and coarctation segment length were set as the main related factors of arterial internal diameter. Complex CoA was defined as CoA with other cardiovascular abnormalities^[19]^.

### Statistical Analysis

IBM Corp. Released 2015, IBM SPSS Statistics for Windows, version 23.0, Armonk, NY: IBM Corp. software was used for statistical analysis. If the measurement data conformed to the normal distribution after the normal test, a *t*-test would be adopted; if they did not conform to the normal distribution, a non-parametric test would be adopted (Mann-Whitney U Test). Pearson’s correlation analysis was used to evaluate the correlation between the *z* value of each aorta and the degree of coarctation. Univariate analysis was used to evaluate possible related factors with ascending aortic dilatation, and multivariate binary logistic regression analysis was used for factors with *P*<0.15 in univariate analysis. The mild CoA group, the severe CoA group, and the VSD group were compared by one-way analysis of variance (ANOVA), and the results of one-way ANOVA were drawn in the form of histogram by GraphPad prism (8.0) software. *P*<0.05 was defined as statistically significant.

## RESULTS

After screening, a total of 75 infantile patients were included in this study, including 21 in the mild CoA group (group A), 26 in the severe CoA group (group B), and 28 in the VSD group (group C). The basic characteristics of the three were shown in Table 1. There were 13 cases of complex CoA in the mild CoA group and 18 cases in the severe CoA group, and the difference between the two was not statistically significant. Among the 47 CoA cases, four cases were complicated with BAV, including two cases in the mild CoA group and two cases in the severe CoA group. In terms of hypertension, there were six cases in the mild CoA group and 13 cases in the severe CoA group. Hypertension in the severe CoA group was more than that in the mild CoA group, but the difference was not statistically significant (*P*=0.117). Through calculation, we found that the severe CoA group had higher aortic stiffness and lower distensibility, but there was no significant difference compared with the mild CoA group and the VSD group (*P*=0.106 and *P*=0.171, respectively) (Table 1).

**Table 1:** General characteristics of the population.

Characteristics	Mild (n=21)	Severe (n=26)	VSD (n=28)	*P*-value
Age (months)	2.74 ± 0.99	2.34 ± 1.33	2.91 ± 1.55	0.294
Male sex (%)	14 (66.67)	18 (69.23)	15 (53.57)	0.447
BSA (m^2^)	0.26 ± 0.11	0.24 ± 0.07	0.30 ± 0.10	0.111
BAV (%)	2 (9.52)	2 (7.69)	-	0.825
Complex CoA (%)	13 (61.90)	18 (69.23)	-	0.413
Hypertension (%)	6 (28.57)	13 (50.00)	-	0.117
CDR	0.60 ± 0.08	0.37 ± 0.07	-	0.000
SBP	75.50 ± 8.35	78.99 ± 7.51	73.74 ± 5.37	0.068
DBP	49.46 ± 8.67	52.39 ± 7.87	48.71 ± 4.81	0.178
LVEF (%)	58.39 ± 5.18	55.54 ± 4.28	57.54 ± 4.00	0.081
Distensibility (mm Hg^-1^·10^-3^)	5.01 ± 0.82	4.77 ± 0.90	5.22 ± 0.90	0.171
Stiffness β index	0.90 ± 0.26	0.97 ± 0.30	0.82±0.20	0.106

BAV=bicuspid aortic valve; BSA=body surface area; CDR=coarctation site-diaphragm ratio; CoA=coarctation of the aorta; DBP=diastolic blood pressure; LVEF=left ventricular ejection fraction; SBP=systolic blood pressure; VSD=ventricular septal defect

The results of CTA measurement showed that ascending aorta was the main dilatation of the aorta in these CoA patients. Among the 47 cases, ascending aorta dilatation was found in 32 cases, descending aorta dilatation was found in 20 cases, pre-coarctation aorta dilatation was found in 17 cases, and aortic arch dilatation was found in six cases. Dilatation of ascending aorta was found in 11 cases and dilatation of descending aorta was found in six cases in the mild CoA group, and dilatation of ascending aorta was found in 21 cases and dilatation of descending aorta was found in 14 cases in the severe CoA group. The difference in dilation rate of ascending aorta between the two groups was statistically significant (11 [52.38%] *vs.* 21 [80.77%], *P*=0.038). Further comparison of the *z* score showed that the *z* score of ascending aorta in the severe CoA group was significantly higher than that in the mild CoA group, and the difference was statistically significant (2.00 ± 0.48 *vs.* 2.36 ± 0.43, *P*=0.010). There was no significant difference in other parts of aortic *z* scores (*P*>0.05). The coarctation segment length in the mild CoA group was 4.78 ± 2.71 mm, which was not significantly different from that in the severe CoA group (6.28 ± 2.83 mm, *P*=0.071) (Table 2). At the same time, we made a pairwise comparison with the VSD group and found that the *z* score of ascending aorta in the severe CoA group was significantly higher than that in the VSD group (*P*=0.000), although the *z* score of ascending aorta in the mild CoA group was also higher than that in the VSD group, but the difference between the two groups was not statistically significant (*P*=0.110) (Figure 1B).

**Table 2:** Comparison of aortic data correlation between mild group and severe group.

Variable	Mild (n=21)	Severe (n=26)	*P*-value
*z* score of aorta			
Ascending aorta	2.00 ± 0.48	2.36 ± 0.43	0.010
Descending aorta	1.87 ± 0.27	2.05 ± 0.33	0.061
Pre-coarctation	1.82 ± 0.33	1.94 ± 0.31	0.206
Aortic arch	1.69 ± 0.25	1.77 ± 0.22	0.244
Aortic dilation (%)			
Ascending aorta	11 (52.38)	21 (80.77)	0.038
Descending aorta	6 (28.57)	14 (53.85)	0.074
Pre-coarctation	5 (23.81)	12 (46.15)	0.138
Aortic arch	2 (9.52)	4 (15.38)	0.554
Length of coarctation	4.78 ± 2.71	6.28 ± 2.83	0.071

Pearson’s correlation analysis was used to test the correlation between related variables and CDR, and it was found that the *z* score of the ascending aorta was negatively correlated with the CDR value (*r*=-0.410, *P*=0.004), while other aortic *z* score, aortic stiffness, and distensibility were not correlated with CDR. Univariate analysis was used to determine the factors related to aortic dilatation. The results showed that age, hypertension, CDR value, and coarctation segment length were correlated with aortic dilatation (*P*<0.15). To reduce collinearity in multivariate analysis, multivariate binary logistic regression analysis was used to correct confounding factors. The study found that only the degree of coarctation (CDR value) was independently related to aortic dilatation (adjusted odds ratio 0.002; 95% confidence interval 0.000~0.819; *P*=0.043) (Tables 3 and 4).

**Table 3:** Correlation between aortic data and degree of coarctation.

Variable	*r*	*P*-value
Ascending aorta	-0.410	0.004
Descending aorta	-0.244	0.098
Pre-coarctation	-0.101	0.500
Aortic arch	-0.100	0.503
Distensibility	0.042	0.781
Stiffness	-0.151	0.310

**Table 4:** Regression analysis of factors related to aortic dilatation.

Univariate binary regression			
Variable	Unadjusted OR	95% CI	*P*-value
Sex	1.278	0.341-4.790	0.716
Age	1.669	0.909-3.064	0.099
Hypertension	0.290	0.068-1.232	0.094
Length of coarctation	1.250	0.969-1.612	0.086
CoA complexity	0.580	0.159-2.112	0.409
Severity of CoA (CDR)	5.562	0.432-0.511	0.023
Multivariate binary logistic regression (adjusted odds)			
Variable	Adjusted OR	95% CI	*P*-value
Age	2.013	0.910-4.452	0.084
Length of coarctation	1.091	0.807-1.473	0.572
Hypertension	0.285	0.057-1.425	0.126
Severity of CoA (CDR)	0.002	0.000-0.819	0.043

CDR=coarctation site-diaphragm ratio; CI=confidence interval; CoA=coarctation of the aorta; OR=odds ratio

## DISCUSSION

CoA is one of the most common CHD^[1-2]^. CoA is usually and simplistically regarded as isolated obstruction of the aortic isthmus, but in fact, isthmic obstruction is only one of many abnormalities which include the dilatation of the proximal and distal aorta, the coronary arteries, conduit arteries (radial, brachial, and carotid), the retinal vascular bed, dissecting aneurysms, cerebral aneurysms, vascular rings, and systemic hypertension^[23]^. In recent years, more and more attention has been paid to aortic dilatation, which is a risk factor for aortic aneurysm and aortic dissection. For patients with CoA, relevant scholars confirmed that there was corresponding aortic dilatation before and after the operation, which was related to the degree of coarctation, age, aortic valve condition, and so on^[5]^. However, which part is the most at risk and what factors affect expansion are still controversial, and the relevant research subjects are mostly adults. Therefore, in this study, we used CTA to evaluate different levels of aortic dilatation and related risk factors in infants with CoA.

This study showed that ascending aorta is often dilated in infantile patients with CoA, and the degree of coarctation is negatively correlated with ascending aortic dilatation. Further regression analysis found that the severity of coarctation was an independent predictor of ascending aortic dilatation. This phenomenon is attributed to the stress difference in the aorta, and the hemodynamic stress related to coarctation may lead to an increase in stroke volume, increasing the wall stress of the ascending aorta, which in turn leads to the dilatation of the ascending aorta^[24]^. Another reason for dilatation of the ascending aorta is that severe coarctation leads to increased afterload of the ascending aorta and increased blood pressure^[24]^. At the same time, we found that some cases were accompanied by a dilatation of descending aorta (lower segment of narrowing), but the difference was not statistically significant, which was different from the results of Zhang^[7]^ and Zhao et al.^[25]^. Zhang’s results showed that with the increase of the degree of coarctation, there was a significant increase in high-intensity vortex structure, accompanied by more intense jet, which led to the dilatation of the lower aorta. The vortex structure with higher vortex intensity gathered downstream of the coarctation segment of the aorta is the decisive factor for the formation of aortic dilatation^[4]^. Zhao et al.^[25]^ believed that this was due to the increase in collateral blood flow and hemodynamic factors caused by the high velocity and turbulent flow downstream of the coarctation, or due to intrinsic character of the aortic wall. We considered that the age of the selected infantile patients was relatively small, the time of vortex blood flow impacting on the wall of the vessel was short, and there was no structural change in the wall of the vessel for a while.

Through TTE, it could be found that the degree of aortic stiffness was higher and the distensibility was poor in infantile patients with severe CoA, but the difference was not statistically significant. Further compared with the degree of CDR, we did not find a significant correlation. The histological results of Sehest et al.^[16]^ showed that collagen and elastin in the aortic wall increased while the content of smooth muscle cells decreased before coarctation. The results of Rogers et al.^[27]^ also showed that age was identified as a risk factor for increased aortic stiffness and might increase the risk of aortic stiffness associated with CoA; the subjects in our study were relatively young, so the results were different from related studies, and further follow-up was necessary in the future. As increased aortic stiffness might lead to hypertension and left ventricular hypertrophy late during follow-up^[27]^, it underscored the need for careful and regular long-term follow-up of patients after CoA repair.

In our study, there was no significant correlation between the five variables and aortic dilatation. Firstly, there was no correlation between the length of the coarctation segment and aortic dilatation, which mainly depended on the narrowest segment. Previous studies had also shown that shorter segments of the aorta were more prone to aortic dilatation^[4]^, which was different from our results. We considered the difference caused by the small sample size and the age, which needed to be further studied in the future. Secondly, there was no correlation between hypertension and aortic dilatation, but although hypertension was generally considered to be a susceptible condition for the development of thoracic aortic aneurysms, the relationship between hypertension and ascending aortic dilatation was still controversial^[27]^. In particular, our study focused on infants, which might lead to different results. Then there was no correlation between age and aortic dilatation. We selected patients younger than one year old as the subjects in this study, which was determined by the actual overall age characteristics of our patients, and there is a certain correlation between age and *z* score^[28]^. Finally, we analyzed the complexity of CoA and found that it was not related to aortic dilatation. We considered that the hemodynamic effect of other intracardiac malformations was much smaller than that of aortic constriction. Many studies had shown that patients with CoA and BAV had more aortic dilatation and confirmed that aortic lesions in the CoA environment were largely caused by BAV^[29]^, while in our 46 cases, there were four cases with BAV, considering less positive variables, so no correlation analysis was carried out.

MSCT has the advantages of fast scanning speed, high spatial resolution, not being affected by heart rate, and low radiation dose, so it has unique advantages in the diagnosis of extracardiac macrovascular malformations^[8,9]^. Aortic rupture is considered to be an important killer of unrepaired patients with CoA, usually developed from aortic dilatation^[5]^. The present results showed that infantile patients with CoA might present with ascending aorta dilatation, and related studies had shown that with increasing age, the ascending aorta progressively dilates and increases the risk of aortic rupture^[30]^. At the same time, our results showed that CoA could promote aortic dilatation, especially in the ascending aorta, which might also aggravate aortic damage after the operation. Therefore, for patients with CoA, CTA scans should be performed routinely to identify the possible risks of aortic dilatation and played an early warning role in clinical treatment. Blais et al.^[31]^ identified uncorrected CoA as a predictor of increased proximal dilatation rate. They believed that only uncorrected CoA was associated with proximal aortic dilatation rate, while corrected CoA did not, suggesting that relief of increased pressure by correction of distal obstruction might slow dilatation rate in our population. Therefore, surgical correction should be performed as soon as possible for infants with CoA, especially those with preoperative dilatation of ascending aorta.

### Limitations

This study was based on a single-center study, and the sample size was relatively limited. To improve the sensitivity and specificity of the study, we may consider collaborating with other centers and collecting more cases in future studies.

## CONCLUSION

Most infants with CoA can also have significant dilatation of ascending aorta, and the degree of this dilatation is related to the degree of coarctation. MSCT can comprehensively evaluate the degree of coarctation in infants with CoA and help to identify the risk of aortic dilatation in patients with CoA.

## References

[1] Kenny D, Hijazi ZM. (2011). Coarctation of the aorta: from fetal life to
adulthood. Cardiol J.

[2] Bower C, Ramsay JM. (1994). Congenital heart disease: a 10 year cohort. J Paediatr Child Health.

[3] Basso C, Boschello M, Perrone C, Mecenero A, Cera A, Bicego D (2004). An echocardiographic survey of primary school children for
bicuspid aortic valve. Am J Cardiol.

[4] Zhang X, Luo M, Fang K, Li J, Peng Y, Zheng L (2020). Analysis of the formation mechanism and occurrence possibility of
post-stenotic dilatation of the aorta by CFD approach. Comput Methods Programs Biomed.

[5] von Kodolitsch Y, Aydin AM, Bernhardt AM, Habermann C, Treede H, Reichenspurner H (2010). Aortic aneurysms after correction of aortic coarctation: a
systematic review. Vasa.

[6] Soleimantabar H, Sabouri S, Khedmat L, Salajeghe S, Memari B, Heshmat Ghahderijani B. (2019). Assessment of CT angiographic findings in comparison with
echocardiography findings of chest among patients with aortic arch
anomalies. Monaldi Arch Chest Dis.

[7] Chen SS, Dimopoulos K, Alonso-Gonzalez R, Liodakis E, Teijeira-Fernandez E, Alvarez-Barredo M (2014). Prevalence and prognostic implication of restenosis or dilatation
at the aortic coarctation repair site assessed by cardiovascular MRI in
adult patients late after coarctation repair. Int J Cardiol.

[8] Rose-Felker K, Robinson JD, Backer CL, Rigsby CK, Eltayeb OM, Mongé MC (2017). Preoperative use of CT angiography in infants with coarctation of
the aorta. World J Pediatr Congenit Heart Surg.

[9] Dionne JM, Abitbol CL, Flynn JT. (2012). Hypertension in infancy: diagnosis, management and
outcome. Pediatr Nephrol.

[10] Pontone G, Baggiano A, Andreini D, Guaricci AI, Guglielmo M, Muscogiuri G (2019). Dynamic stress computed tomography perfusion with a whole-heart
coverage scanner in addition to coronary computed tomography angiography and
fractional flow reserve computed tomography derived. JACC Cardiovasc Imaging.

[11] Papadakis AE, Damilakis J. (2021). Technical note: quality assessment of virtual monochromatic
spectral images on a dual energy CT scanner. Phys Med.

[12] Dijkema EJ, Slieker MG, Leiner T, Grotenhuis HB. (2018). Arterioventricular interaction after coarctation
repair. Am Heart J.

[13] Update on the 1987 task force report on high blood pressure in
children and adolescents: a working group report from the national high
blood pressure education program (1996). National high blood pressure education program working group on
hypertension control in children and adolescents. Pediatrics.

[14] Borow KM, Newburger JW. (1982). Noninvasive estimation of central aortic pressure using the
oscillometric method for analyzing systemic artery pulsatile blood flow:
comparative study of indirect systolic, diastolic, and mean brachial artery
pressure with simultaneous direct ascending aortic pressure
measurements. Am Heart J.

[15] Nabati M, Namazi SS, Yazdani J, Sharif Nia H. (2020). Relation between aortic stiffness index and distensibility with
age in hypertensive patients. Int J Gen Med.

[16] O'Rourke MF, Staessen JA, Vlachopoulos C, Duprez D, Plante GE. (2002). Clinical applications of arterial stiffness; definitions and
reference values. Am J Hypertens.

[17] Becker C, Soppa C, Fink U, Haubner M, Müller-Lisse U, Englmeier KH (1997). Spiral CT angiography and 3D reconstruction in patients with
aortic coarctation. Eur Radiol.

[18] Robicsek F. (1955). Post-stenotic dilatation of the great vessels. Acta Med Scand.

[19] Türkvatan A, Akdur PO, Olçer T, Cumhur T. (2009). Coarctation of the aorta in adults: preoperative evaluation with
multidetector CT angiography. Diagn Interv Radiol.

[20] Colan SD, McElhinney DB, Crawford EC, Keane JF, Lock JE. (2006). Validation and re-evaluation of a discriminant model predicting
anatomic suitability for biventricular repair in neonates with aortic
stenosis. J Am Coll Cardiol.

[21] Dallaire F, Bigras JL, Prsa M, Dahdah N. (2015). Bias related to body mass index in pediatric echocardiographic Z
scores. Pediatr Cardiol.

[22] Lindahl S, Okmian L. (1981). Bedside calculation of body surface area for infants and
children. Crit Care Med.

[23] Perloff JK. (2010). The variant associations of aortic isthmic
coarctation. Am J Cardiol.

[24] Tadros TM, Klein MD, Shapira OM. (2009). Ascending aortic dilatation associated with bicuspid aortic
valve: pathophysiology, molecular biology, and clinical
implications. Circulation.

[25] Zhao Q, Shi K, Yang ZG, Diao KY, Xu HY, Liu X (2018). Predictors of aortic dilation in patients with coarctation of the
aorta: evaluation with dual-source computed tomography. BMC Cardiovasc Disord.

[26] Sehested J, Baandrup U, Mikkelsen E. (1982). Different reactivity and structure of the prestenotic and
poststenotic aorta in human coarctation. Implications for baroreceptor
function. Circulation.

[27] Rogers WJ, Hu YL, Coast D, Vido DA, Kramer CM, Pyeritz RE (2001). Age-associated changes in regional aortic pulse wave
velocity. J Am Coll Cardiol.

[28] Elkinany S, Weismann CG, Curtis A, Smith T, Zafar MA, Breen T (2021). Is aortic z-score an appropriate index of beneficial drug effect
in clinical trials in aortic aneurysm disease?. Am J Cardiol.

[29] Beaton AZ, Nguyen T, Lai WW, Chatterjee S, Ramaswamy P, Lytrivi ID (2009). Relation of coarctation of the aorta to the occurrence of
ascending aortic dilation in children and young adults with bicuspid aortic
valves. Am J Cardiol.

[30] Luijendijk P, Franken RJ, Vriend JW, Zwinderman AH, Vliegen HW, Winter MM (2013). Increased risk for ascending aortic dilatation in patients with
complex compared to simple aortic coarctation. Int J Cardiol.

[31] Blais S, Meloche-Dumas L, Fournier A, Dallaire F, Dahdah N. (2020). Long-term risk factors for dilatation of the proximal aorta in a
large cohort of children with bicuspid aortic valve. Circ Cardiovasc Imaging.

